# Interface and Interphase in Polymer Nanocomposites with Bare and Core-Shell Gold Nanoparticles

**DOI:** 10.3390/polym13040541

**Published:** 2021-02-12

**Authors:** Albert J. Power, Ioannis N. Remediakis, Vagelis Harmandaris

**Affiliations:** 1 Department of Mathematics and Applied Mathematics, University of Crete, GR-71409 Heraklion, Crete, Greece; 2 Institute of Applied and Computational Mathematics (IACM), Foundation for Research and Technology Hellas (FORTH), GR-71110 Heraklion, Crete, Greece; 3 Department of Materials Science and Technology, University of Crete, GR-71003 Heraklion, Crete, Greece; remed@materials.uoc.gr; 4 Institute of Electronic Structure and Laser, (IESL), Foundation for Research and Technology Hellas (FORTH), GR-71110 Heraklion, Crete, Greece; 5 Computation-Based Science and Technology Research Center, The Cyprus Institute, Nicosia 2121, Cyprus

**Keywords:** molecular dynamics simulations, gold, nanoparticles, core-shell, grafted, structural and dynamical properties of polymers, polyethylene

## Abstract

Metal nanoparticles are used to modify/enhance the properties of a polymer matrix for a broad range of applications in bio-nanotechnology. Here, we study the properties of polymer/gold nanoparticle (NP) nanocomposites through atomistic molecular dynamics, MD, simulations. We probe the structural, conformational and dynamical properties of polymer chains at the vicinity of a gold (Au) NP and a functionalized (core/shell) Au NP, and compare them against the behavior of bulk polyethylene (PE). The bare Au NPs were constructed via a systematic methodology starting from ab-initio calculations and an atomistic Wulff construction algorithm resulting in the crystal shape with the minimum surface energy. For the functionalized NPs the interactions between gold atoms and chemically adsorbed functional groups change their shape. As a model polymer matrix we consider polyethylene of different molecular lengths, from the oligomer to unentangled Rouse like systems. The PE/Au interaction is parametrized via DFT calculations. By computing the different properties the concept of the interface, and the interphase as well, in polymer nanocomposites with metal NPs are critically examined. Results concerning polymer density profiles, bond order parameter, segmental and terminal dynamics show clearly that the size of the interface/interphase, depends on the actual property under study. In addition, the anchored polymeric chains change the behavior/properties, and especially the chain density profile and the dynamics, of the polymer chain at the vicinity of the Au NP.

## 1. Introduction

The study of polymer-based hybrid materials is a field of immense interest as it involves a broad spectrum of systems, applications, and spatiotemporal scales. On polymer/solid nanostructured systems in particular, the solid phase can strongly modify the properties of the entire hybrid system, such as its mechanical and electrical ones, as well as its dynamical/rheological behavior [1,2,3,4,5,6,7]. Therefore, the investigation of model polymer/solid interfacial systems, at the molecular level, is an intense research area, since such interfaces play a crucial role on the behavior of polymer-based systems with important technological applications, including for example polymer nanocomposites, polymer coatings, lubricants and thin films [8,9,10,11,12]. Examples include the modification of the electrochemical behavior [13] and the amelioration of the thermal degradation of the nanocomposites [14]. Moreover, there have been reports of enhancement of hardness, solvent resistance and glossiness of nanocomposites [15]. The improvement of the tensile strengths of nanocomposite films [16] and the enhancement of the interfacial adhesion between nanoparticle and polymer matrix are also very important [17]. Furthermore, nanoparticles modify the mechanical properties of a polymer matrix [18,19].

From the broad family of polymer nanocomposites (PNCs) here we focus on systems with (bare and core/shell) metal nanoparticles (NPs). Such systems have been used in the recent past in bio-nano-technology for biomedical utilization, including antibacterials [20], antimicrobials [21,22], biosensors [23], cancer treatment [24] and biomedical tissue engineering [25,26]. Their usage is also explored in other technological applications involving catalytic devices, in the textile industry and in food packaging [27,28,29,30,31,32]. In particular, polymer systems with dispersed gold (Au) NPs, or core/shell gold NPs, have been extensively studied due to their exceptional properties, such as biocompatibility, tunable conductivity and catalytic properties. Au nanoparticles of few nanometers (1 to 100 nm) have a great surface/volume ratio and that enables their surface to be coated with many molecules (including therapeutics and targeting agents). Moreover, they are among the most stable of metal nanoparticles and they also provide a stable immobilization platform of the molecules while their reactivity is conserved. Their properties include colloidal stability and the ability to be conjugated with ease with biological molecules. Applications of polymer nanocomposites with Au NPs span many scientific fields, such as medicine [33] biotechnology [34], catalysis [35], and electronics [36]. In all these applications, the shape of Au nanoparticles has a key role in every aspect of their functionality, from sensing [37] and biolabeling applications [38] to plasmonics [39], photonics [40] and fuel cells [41]. Additional technological areas in which gold nanoparticles have been used include: the storage of energy [42], the delivery of molecules into cells [33,43], use as a heat source [44], as sensors [45,46,47], labeling [48,49], Light Emitting Diode (LED) applications [50], optical and electronic applications [39], drug delivery vehicles [51,52] and in the field of catalysis [35,53,54,55].

Besides experiments [56,57,58,59,60,61,62,63,64], molecular simulations have been used to study the properties of polymer-based complex materials [65,66,67,68,69,70,71], including atomistic molecular dynamics (MD) [3,72,73,74,75,76], dynamic Monte Carlo simulations (MCMD) [77,78,79], self-consistent field theory (SCFT) and density functional theory (DFT) [80], dissipative particle dynamics (DPD) [81], coarse-grained (CG) MD simulations [82,83,84,85] and stochastic dynamics simulations [86].

We should note that polymer/NP hybrid system have been already simulated in the past, mainly via qualitative CG bead spring models, but atomistic simulations as well. However, limited are the atomistic works including metals, and especially Au, NPs. Given the important role of the actual chemistry on the polymer/NP interaction and on the overall properties of the hybrid material, quantitative atomistic simulations of specific systems can be valuable tools, complementary to experiments, to provide a fundamental study of specific systems.

Here we study via atomistic simulations polymer nanocomposites with bare and grafted Au NPs; the latter were constructed in their minimum energy configuration, via the Wulff method, whereas the PE/Au interaction is derived via DFT calculations. We further examined and compared hybrid systems with bare and grafted Au NPs, focusing on the PE/Au interphase. Moreover, properties of polymer chains are studied as a function of: (a) size of the Au NP, (b) MWs of the polymer matrix and (c) lengths of the anchored polymeric chains.

It is now acknowledged that the behavior of polymer chains close to a polymer/solid interface is different from the behavior of the bulk [87,88,89,90,91,92]. For such systems, an interphase between the substrate and the bulk phase of the polymer is postulated, and the width of this interphase layer has been the focus of many studies. For example, it has been observed that segmental packing and orientation return to bulk values within just a few segment lengths from the surface and chain properties reached the bulk values after a few 1–2 times the radius of gyration *R_g_* using atomistic and systematic coarse-grained models [88,93], or bead–spring models [94,95]. In addition, concerning the segmental dynamics of the macromolecules, relaxation times of segments at the vicinity of a solid surface strongly depend on the strength of the polymer/surface interactions [89,96]. For polymer chains supported by a solid substrate the size of the interface or interphase depends on the actual property under study [88].

Furthermore, coarse-grained MD, Monte Carlo MD and atomistic MD simulations have been used to examine the viscoelastic behavior and the dispersion−aggregation transition of NPs in polymer nanocomposites with polymer-grafted nanoparticles systems [97,98,99,100,101], to compute the mean square displacement and the mean relaxation time of various intramolecular vectors [102], the structural properties and the mass density profiles of polymers brushes (grafted) [67,78]. Moreover, several studies have investigated the polymer’s structure, rheological properties and the shearing of the polymer between two gold surfaces, using MD simulations [103,104,105]. Finally, the mass density profiles, the mean square displacement, the end to end distance and the radius of gyration of polymer chains for PNCs with gold nanoparticle have been examined through MD simulations as well [73].

Despite the above works, the study of the polymer/metal NP interface, and interphase as well, by predicting quantitatively the properties of polymer chains of specific polymer nanocomposites, using realistic atomistic models for both polymer matrix and the Au NP is still a challenging problem. The main goal of this work is to provide a detailed investigation of polymer nanocomposites with dispersed gold nanoparticles and core/shell gold nanoparticles, at the molecular level through detailed MD simulations. As model polymer we consider polyethylene (PE). Bulk PE and PE-based nanocomposite materials are among the most widely used polymers in industry, and have been studied in depth during the recent years through experiments [106,107,108] and simulations [96,109,110]. The Au NPs and the functionalized Au NPs are made with Wulff construction derived directly from density functional theory (DFT) calculations [111,112] in order to obtain model Au NPs with the minimum surface free energy, i.e., at thermodynamic equilibrium.

In the next section the model, the simulation method and the analysis techniques that we have used are described. Our results, divided in subsections, are presented in Section 3. Finally, a summary and the conclusions of the current study are presented in Section 4.

## 2. Model and Simulation Method

We study PE nanocomposites with bare Au NPs and with functionalized, with short PE chains, (core/shell) NPs. The model gold nanoparticles, were generated using an atomistic Wulff construction algorithm [111,112]. The grafting of the gold nanoparticles was accomplished by using anchored polyethylene chains. In all cases the temperature is 450 K, above the melting temperature of PE. Ten (10) different model systems are considered, involving two different monodisperse PE matrices (Table 1); one consists of chains with 22 monomers (MW = 310 gr/mol) and the other one with 100 monomers (MW = 1400 gr/mol) [109,110,113]. Two different gold NP sizes with Wulff construction were modeled [111,112]: one with diameter of 25 Å and one with 50 Å. Systems with the same polymer matrix but a different nanoparticle are also studied. Both of the grafted Au nanoparticles have a diameter of 5 nm and 53 grafted polyethylene chains. The first one has 20 monomers per chain and the other one 62 monomers per chain. The grafting density is 0.67 chains per square nm. That value has been used in several experimental studies of different PNCs [114]. The equilibrium shape of nanoparticles is a polyhedron that is derived through the Wulff construction. Small nanoparticles often deviate from this thermodynamically stable equilibrium shape: for example, at small diameters, an edge length might be smaller than the atom diameter. For Au, the smallest nanoparticle that has same polyhedral shape as the Wulff construction has a diameter of about 2.5 nm for both bare- and thiol-covered nanoparticles [111,112]. For comparison, we also considered larger nanoparticles with diameters around 5 nm.

**Figure 1:**
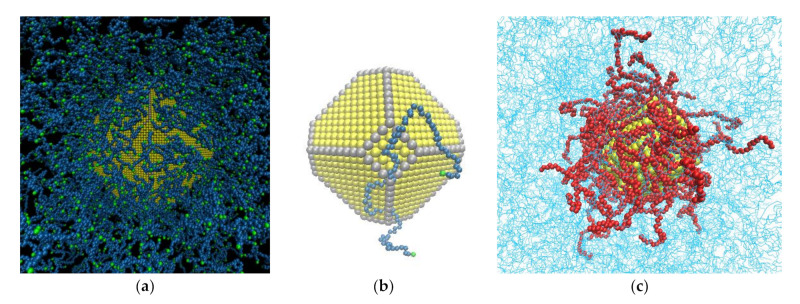
Snapshots from the model systems: (**a**) The PE22/Au5 hybrid system. In yellow is the Au, in blue are the CH_2_ monomers and in green are the CH_3_ monomers. (**b**) Au NP, of *d* = 5.02 nm (in yellow are the Au atoms and in grey the edge Au atoms), and a PE 100 mer chain. (**c**) The PE100/Au5/g62 hybrid system. In blue are the free CH_2_ and the free CH_3_ monomers. In red are the grafted CH_2_ and CH_3_ monomers.

**Figure A1:**
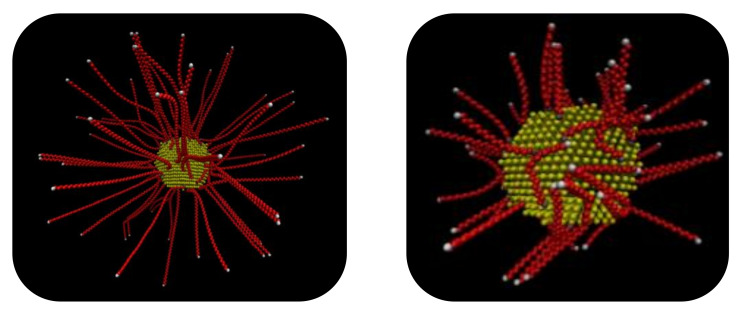
Snapshot from MD simulation of hybrid polyethylene/grafted gold nanoparticle at 450 K. Au nanoparticle (2461 atoms, diameter of 5.04 nm) is shown. In yellow is the Au and in grey are the terminal CH_3_ groups. In red are the grafted CH_2_ and the grafted CH_3_ monomers. The initial configuration of the grafted NP with the short anchored polymeric chains and with the long anchored polymeric chains.

More details for all systems are presented in Table 1. Typical snapshots of the model PE/Au (bare and core/shell) NP systems are shown in Figure 1 and in Appendix A Figure A1. A video (Supplementary Materials) from our MD simulation of hybrid polyethylene/grafted gold nanoparticle at 450 K is available online at www.mdpi.com/2073-4360/13/4/541/s1, Video S1: PE-grafted AuNP.

We used the LAMMPS package [115] to perform Molecular Dynamics (MD) simulations in the isothermal-isobaric (NPT) statistical ensemble. We kept the pressure constant at *P* = 1 atm by using Nosé Hoover barostat. To keep the temperature at *T* = 450 K we used the Nosé Hoover thermostat. Periodic boundary conditions in all three dimensions were used, whereas the integration time step was 1.0 fs. Furthermore, we used a united atom model to represent the polyethylene. In this case, each methylene CH_2_ and methyl CH_3_ group represented as a single Van der Waals interacting site. Harmonic potential was used to describe the polyethylene bonds and angles whereas the OPLS force field (Appendix A Table A1) was used to describe the polyethylene dihedrals. For the Van der Waals interactions between the PE-PE (Appendix A Table A2) we used a spherically truncated 6–12 Lennard–Jones potential with cutoff distance *R*_c_ *=* 10 Å [109]. The first gold nanoparticle with Wulff construction has 459 atoms with 2.51 nm diameter and the second has 3101 atoms with 5.02 nm diameter [111,112]. The interaction between the Au and PE is described via a Morse potential, which is taken from the literature and is based on detailed DFT calculations [88,93,116]. This potential is parametrized in order to describe with accuracy extensive DFT data regarding the adsorption energy of the ethylene on the Au surface as a function of distance for several different adsorption sites.

For the core/shell Au NP systems the S atoms were placed on the Au NPs in their minimum energy positions as computed from the DFT calculations [116]. Interactions between the S and CH_x_ groups of PE were modeled via a 6–12 Lennard–Jones potential with cutoff distance *R*_c_ *=* 10 Å (see Table A2 in Appendix A). For the S–CH_2_–CH_2_–CH_2_ dihedral angle interactions the OPLS force field was used. The entire atomistic force field is given in Table A1 and Table A2 of the Appendix A. Tail corrections were applied to both energy and pressure. For the non-bonded interactions between PE-PE monomers, the Lorentz–Berthelot rules were used. The gold nanoparticles are frozen during the duration of the MD runs. This is not expected to be a crude assumption since the Au NPs are very stable under conditions (temperature and pressure) similar to those of the current simulations.

### 2.1. Shape of Au NPs

Gold nanoparticles can be found in nature in various shapes. Here we consider their “equilibrium” conditions, i.e., the shape with the minimum surface energy [53,54,112,117]. At the limit of very large nanoparticles (thermodynamic limit), the shape is a polyhedron. The faces of this polyhedron are parallel to different (*hkl*) orientations of the crystal lattice. The total surface energy, *F*, of the nanoparticle, given by,

(1) $$F=\;\underset{hkl}{\sum }A_{hkl}\gamma_{hkl}$$

is minimum, where the summation is over all faces of the nanoparticle. $A_{hkl}$ is the sum of areas of all faces that are parallel to the (*hkl*) crystal plane and $\gamma_{hkl}$ is the surface tension of the material (energy per unit area) when its surface is cleaved parallel to the (*hkl*) plane of the crystal.

G. Wulff proposed that the shape that minimizes surface energy in Equation (1) is such that the distance of each face from the center is proportional to the surface tension of the respective (*hkl*) surface [118]:

(2) $$d_{hkl}∼\gamma_{hkl}$$

The surface tensions, $\gamma_{hkl}$, can be calculated by simulating thick enough slabs of the material with faces parallel to the *(hkl)* crystal plane. Typically, the total energy is calculated using quantum-mechanical calculations, such as Density-Functional Theory (DFT). The equilibrium shapes of gold nanoparticles used in the present work were obtained by coupling extensive DFT calculations to Wulff constructions [111,112]. Through this hybrid methodology atomistic models of nanoparticles were constructed with diameters up to several tenths of a nanometer [118,119,120].

Different (*hkl*) planes bind differently to functional groups due to the presence of different atomic arrangements. As a result, the energies $\gamma_{hkl}$ depend not only on the orientation (*hkl*) but also on the binding energy and the grafting density on the surface. The binding energies can be calculated using DFT [117]. This multi-scale scheme has been used to analyze shapes of nanoparticles of different materials, including SiO_2_ [120], Au with adsorbed CO [112], Ag [121], and Pt in HCl [122].

Here, we use the nanoparticles generated with the method used in Ref. [111]. In that work, DFT and Wulff construction was used to calculate equilibrium shape of gold with adsorbed alkanethiols. The resulting shape resembled a deltoidal icositetrahedron with twenty-four faces oriented towards (211). In the absence of alkanethiols, the equilibrium shape was a truncated octahedron, with eight (111) faces and six (100) faces. Smaller nanoparticles usually have small deviations from the Wulff equilibrium shape as the smallest faces might have areas comparable to the atom cross-sections.

### 2.2. Generation and Equilibration of Model Systems

Generation and equilibration of model polymer nanocomposites is not a trivial issue. Below we describe shortly the procedure followed in order to obtain the model PE/Au nanocomposites:

(a) First, in order to obtain initial PE/grafted Au configurations, we added the anchors to the Au surface randomly by using a Monte Carlo algorithm in suitable positions according to the shape of the Au and taking into account the absorption sites of sulfur in the DFT calculations of alkanethiols adsorbed on Au.
(b) Second, we equilibrate the hybrid system through energy minimization and long simulation runs. Energy minimization of the core/shell system was performed followed by MD simulation runs up to 10 ns in the NVT ensemble. Then, the Au nanoparticle, grafted or not, was placed at a close distance (about 0.5 nm) to several well-equilibrated polymer samples [109].
(c) The final step of our “equilibration protocol” involves the execution of long MD simulations, of the order of 30 ns. Throughout this time we monitored the motion of the whole hybrid system. Our simulations run times were much higher than the relaxation times of the chains [109].

We used various criteria to ensure equilibration of the model systems. We computed the time evolution of the radius of gyration, *R*_g_, values and checked the de-correlation of the end to end vector (ACF) of polymer chains. Furthermore, we performed several (3–5) different simulations by following the exact same procedure but starting with different initial configurations and we end with the same results.

Starting from the well equilibrated atomistic PE/Au configurations, we executed production runs for times up to 100 ns. We saved many thousands of PE/Au NP configurations. We used these configurations to estimate the properties of the whole hybrid systems and for the detailed analysis of PE/NP interfaces. Note, that the above methodology can be expanded to provide well equilibrated atomistic configurations of other polymer/core shell NP nanocomposites as well.

### 2.3. Analysis Method

**Figure 2:**
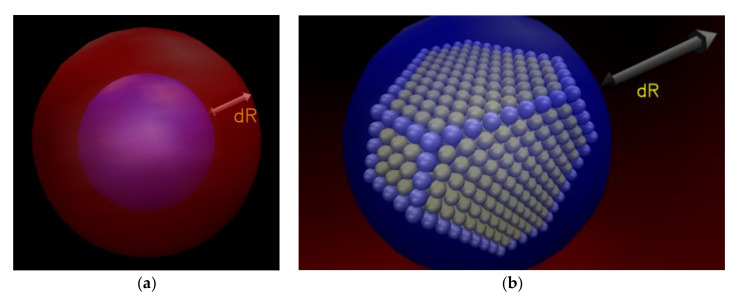
(**a**) A sketch of the analysis scheme in spherical shells. (**b**) Inside view of the Figure 2a.

Our main goal is to study the spatial and dynamical heterogeneities of model hybrid polymer/nanoparticle systems in a detailed way at the molecular level. Consequently, we examined properties of the polymer chains as a function of the distance from the Au NP [96,119]. This analysis was achieved by forming spherical shells of increasing radius (i.e., increasing distances form the Au NP), for each saved configuration, along radial distances from the center of mass of the gold NP (see Figure 2).

Subsequently, we calculated the mass density profiles based on the above radial distance analysis. The thickness of the spherical shells involved was 1 Å. To calculate the second rank bond order parameter we used the same binning (thickness of spherical shells). To calculate the orientational and translational dynamical properties in segmental level and to improve statistics we used spherical shells of a thickness of around 5–10 Å. For the calculation of the mean squared displacement we used spherical shells of a thickness of around 15–20 Å. We chose the binning size for the computation of each specific property based on an optimal combination of detailed information and improved statistics. Furthermore, for the calculation of the density of PE as a function of the distance from the nanoparticle, the mass (computed via the number) of PE atoms within each spherical shell was divided by its volume.

## 3. Results

### 3.1. Structural Properties

We start the analysis of the simulation results by investigating the structural properties of the model atomistic PE/Au nanocomposites.

#### 3.1.1. Density Profiles

**Figure 3:**
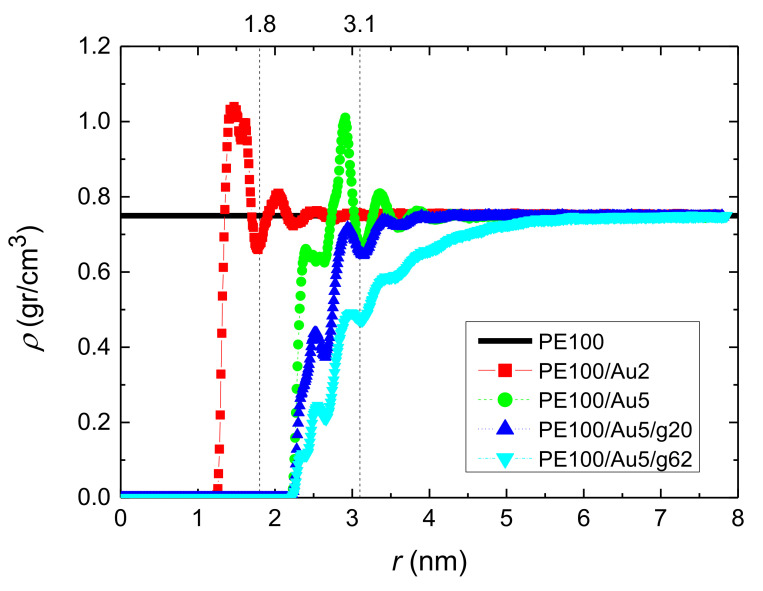
Mass monomer density profiles of polyethylene as a function of *r* (distance from the center of the gold NP) for the systems: PE100, PE100/Au2, PE100/Au5, PE100/Au5/g20 and PE100/Au5/g62.

**Figure A2:**
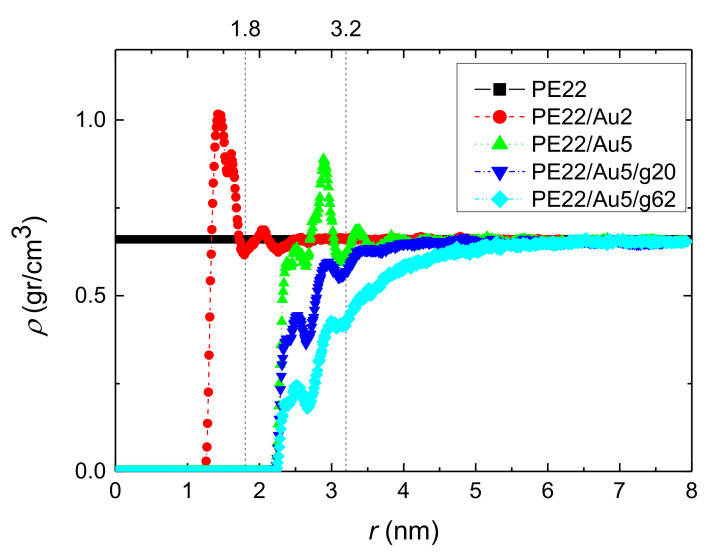
Mass monomer density profiles of PE chains as a function of distance from the center of the gold NP, *r*, for the systems: PE22, PE22/Au2, PE22/Au5, PE22/Au5/g20 and PE22/Au5/g62.

The analysis of the PE/Au model nanocomposites was commenced by calculating the mass monomer density profile of the polymer (PE) chains as a function of the distance from the gold NP. All systems consisting of polyethylene matrices of 100 mers per chain whose average density profiles we calculated for the center of mass of the monomers, *ρ(r)*, are shown in Figure 3 and all systems consisting of PE matrices of 22 mers per chain are shown in Appendix A Figure A2.

In Figure 3, the polymer mass at each spherical shell has been divided by the total volume of the shell. Far from the Au NP, all curves reach/approach the bulk density value (*ρ* = 0.75 gr/cm^3^), though at different distances due to the different Au NP sizes. PE100/Au2 and PE100/Au5 systems exhibit the same behavior: a peak of rather similar height (but larger than the bulk value) is observed at a distance/radius of about 1.3 nm and 1.8 nm respectively, which denotes the attraction of the polymer atoms from the gold NP at short distances, due to vdW forces, while at longer distances the bulk density is attained. In the core/shell Au NP systems (PE100/Au5/g20 and PE100/Au5/g62), only few polyethylene chains can penetrate the anchors and reach the gold surface. We observe a similar behavior for the systems consisting of PE matrices of 22 mers per chain although in this case the average density is lower than that of the systems consisting of PE matrices of 100 mers per chain. The above values are in very good agreement with experimental data for bulk PE chains [123].

**Figure 4:**
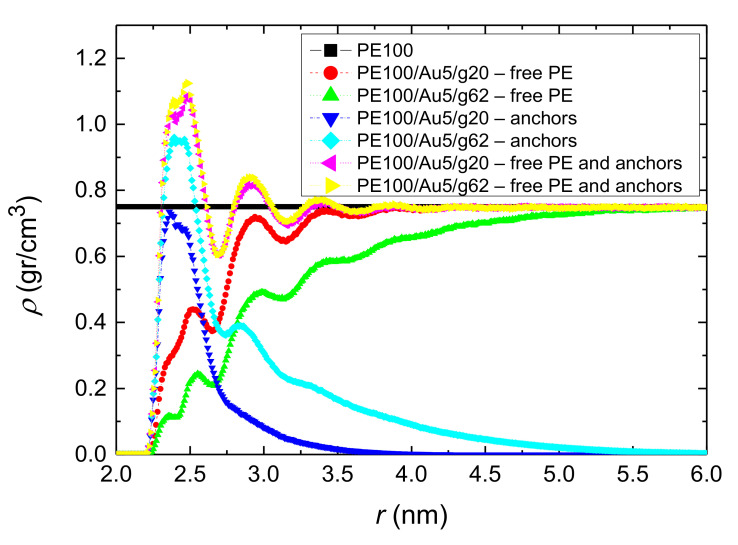
Mass monomer density profiles of polyethylene as a function of *r* (distance from the center of the gold NP). Au5/g20 and PE100/Au5/g62 systems. The density profile was decomposed to free polyethylene chains and grafted polyethylene chains.

For the core/shell NP systems, the density profile can be decomposed to free polyethylene chains and grafted polyethylene chains. As free PE chains we consider the PE matrix and as grafted PE chains, the grafted chains that are anchored on the gold NP. In Figure 4, the total PE density profiles is shown as well as its decomposition in “free” and “grafted” chains. We observe that the density values for the free polyethylene chains are lower than the corresponding bulk value close to the surface due to the nanoparticle’s anchors, which do not allow the interpenetration. However, the NP with short anchors allows more free PE chains to reach close to the surface compared to the case of long anchors NP. On the other hand for the grafted polyethylene chains (i.e., PE100/Au5/g20 and PE100/Au5/g62 systems) we observe a peak close to the Au NP due to the attraction from the surface. This peak is more pronounced for the case of PE100/Au5/g62 system due to the longer anchors. Moreover, the extension of anchors is up to 35 Å and up to 55 Å for the PE100/Au5/g20 and PE100/Au5/g62 systems respectively. Therefore, the corresponding bulk values are attained at these distances, as is observed in the density profiles of the total density curves (sum of free and grafted polyethylene chains). A similar behavior is observed for the PE22/Au2, PE22/Au5, PE22/Au5/g20 and PE22/Au5/g62 systems however in this case the average density is lower than that of the systems consisting of PE matrices of 100 mers per chain.

#### 3.1.2. Structure of PE Chains

**Figure A3:**
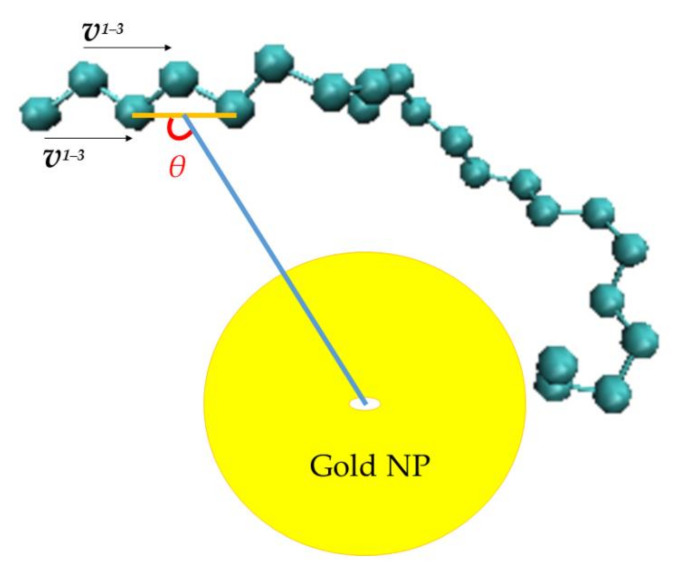
The definition of the *θ* angle for the calculation of the second rank bond order parameter *S*_1–3_ of polyethylene chains for ***v***^1−3^ vector. In blue is the PE and in yellow is the Au NP. The orange line connects two non-consecutive carbon atoms and the blue line connects the center of the gold NP with the midpoint of the orange line. In red is the *θ* angle.

Bellow we examine the orientation of the polymer chains close to the gold NP in the segmental level, through the ***v***^1−3^ vector, which connects two non-consecutive carbon atoms. The segmental orientation is quantified via the second rank bond order parameter [12,124] defined as:

(3) $$S_{1-3}=\frac{3}{2}\langle \cos^{2}\theta \rangle -\frac{1}{2}$$

where *θ* is the angle between a vector which is defined along the chain (here the ***v***^1−3^ one) and one that connects the center of the gold NP with the midpoint of the above (***v***^1−3^) vector (see Figure A3 in Appendix A), and whereas brackets 〈 〉 denote statistical average. *S*_1–3_ limiting values of −0.5, 0.0, and 1.0 correspond to perfectly parallel, random, and perpendicular vector orientations relative to the Au NP, respectively. For the limiting values we assume smooth plain surface.

**Figure 5:**
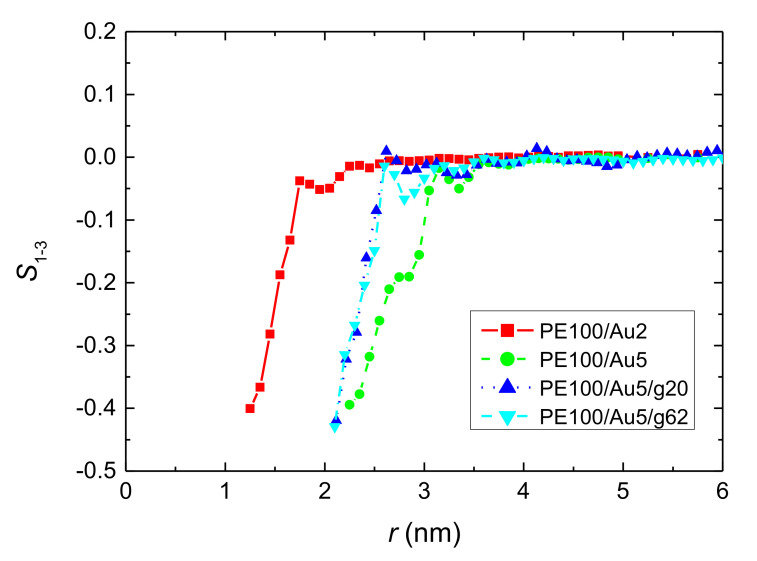
Second rank bond order parameter *S*_1–3_ of polyethylene chains for ***v***^1−3^ vector, as a function of distance from the center of the Au NP, *r*, for all PE/Au systems with PE matrices consisting of 100 mers per chain.

The bond order parameter of ***v***^1−3^ for all systems with PE matrices consisting of 100 mers per chain is depicted in Figure 5. In all cases there is an obvious tendency of the segments of the polymer chain for an almost parallel orientation relative to the Au NP surface at short distances which is gradually randomized the further the distance. There is a decrease of the bond order parameter of the PE segments closest to the Au NP and the minimum values are about −0.4 for all hybrid systems. The same behavior is observed for the other model systems studied here as well.

**Figure 6:**
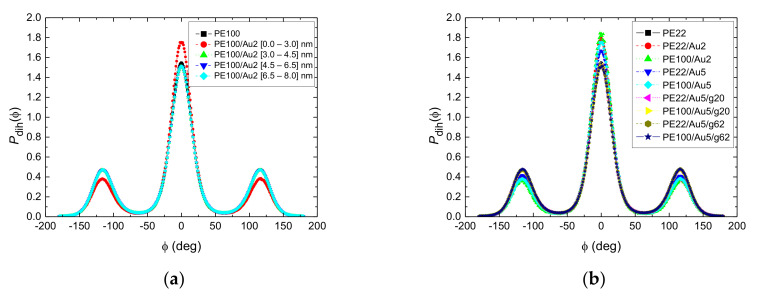
(**a**) Torsional angles distribution of PE chains for various distances from the center of the gold NP for the PE100/Au2 system and the corresponding PE bulk curve. (**b**) Torsional angles distributions of all model systems for PE chains belonging in the first adsorbed layer, i.e., being closer to the Au NP. The corresponding curves for bulk PE are also shown.

To further analyze the PE chain conformations at the segmental level we probe the distribution of the torsional (dihedral) angles, *P*_dih_, of polymer chains at different distances from the gold NP. Results about the dihedral angle distributions of the PE chains are shown in Figure 6a for the PE100/Au2 system (“trans” corresponds to 0°, “gauche-” and “gauche+” to −60*°* and +60° respectively and “cis” to 180° degrees). For the first adsorption layer, defined via the first minimum in the density profile (0–30 Å, see Figure 3), a non-negligible enhancement of the trans states with a consequent reduction of the gauche ones is observed for PE22/Au2, PE22/Au5, PE100/Au2 and PE100/Au5 systems compared to the bulk case (Figure 6b). This observation reflects the more ordered PE chains close to the gold NP. Enhancement of “trans” population would be expected to affect the crystallinity of PE chains as well as the mechanical properties of the hybrid system. Such a behavior has been observed for PE adsorbed on planar carbon-based surfaces, such as graphite or graphene, where the structure of PE commensurate to the underlying crystal structure of the substrate [3,96,125,126]. Here the enhancement of “trans” population is rather weak.

Concerning the system with the functionalized Au NPs (PE22/Au5/g20, PE22/Au5/g62, PE100/Au5/g20 and PE100/Au5/g62) no differentiation in the torsional angle distributions is detected. Short anchors as in PE100/Au5/g20 and PE22/Au5/g20 systems are enough to make the dihedral distribution peak to disappear. For the most distant adsorption layer (i.e., bulk region), the curves are completely identical to each other and to the corresponding bulk one.

**Figure A4:**
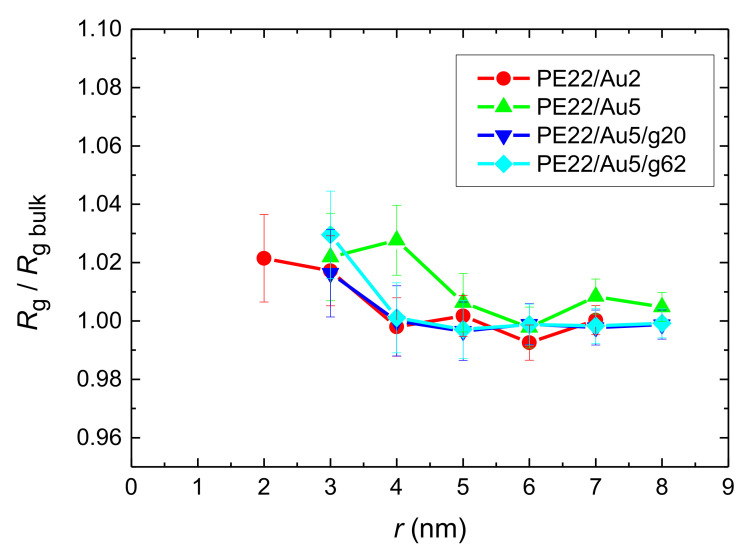
Radius of gyration of PE chains, scaled with its bulk value (*R*_g/_*R*_g bulk_) as a function of *r* distance from the center of the Au NP. Data for the PE22, PE22/Au2, PE22/Au5, PE22/Au5/g20 and PE22/Au5/g62 systems are shown.

**Figure A5:**
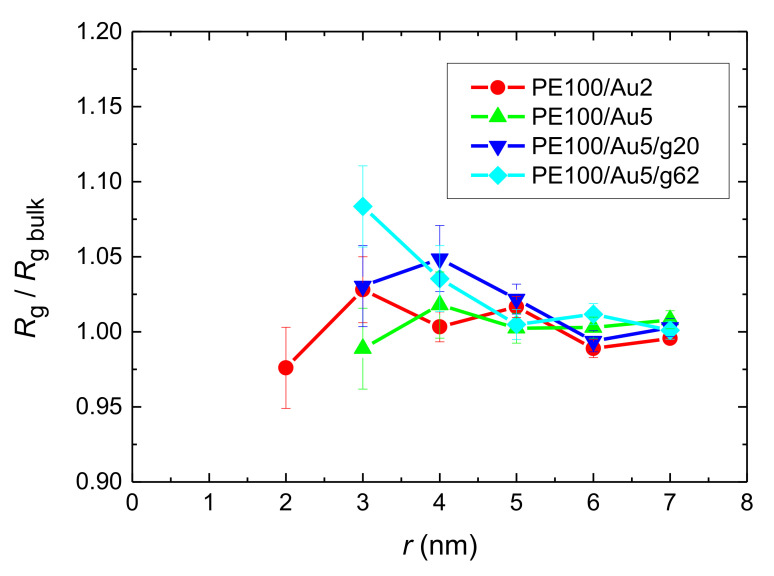
Radius of gyration of PE chains, scaled with its bulk value (*R*_g*/*_*R*_g bulk_) as a function of distance, *r*, from the center of the gold NP. Data for the PE100, PE100/Au2, PE100/Au5, PE100/Au5/g20 and PE100/Au5/g62 systems are shown.

In addition, the radius of gyration (*R*_g_) for the PE was calculated and found approximately 6 Å in the systems consisting of 22 monomers per chain (Appendix A Figure A4) and approximately 16 Å in the systems consisting of 100 monomers per chain (Appendix A Figure A5). These values are very close to the experimental data [127]. Moreover, we observed a small increment, about 5%, of the *R*_g_ close to the surface area, as we expected. Such perturbation of the *R*_g_ has been also observed in other polymer nanocomposite systems as for example PE with graphene [96].

### 3.2. Dynamical Properties

In this section we present data concerning the dynamical properties of polymer chains in the model nanocomposites with bare, and functionalized core/shell Au NPs. We perform the analysis by calculating corresponding quantities of PE chains, both as averages for the entire nanocomposite and as a function of distance from the PE/Au interface.

#### 3.2.1. Orientational Dynamics

**Figure 7:**
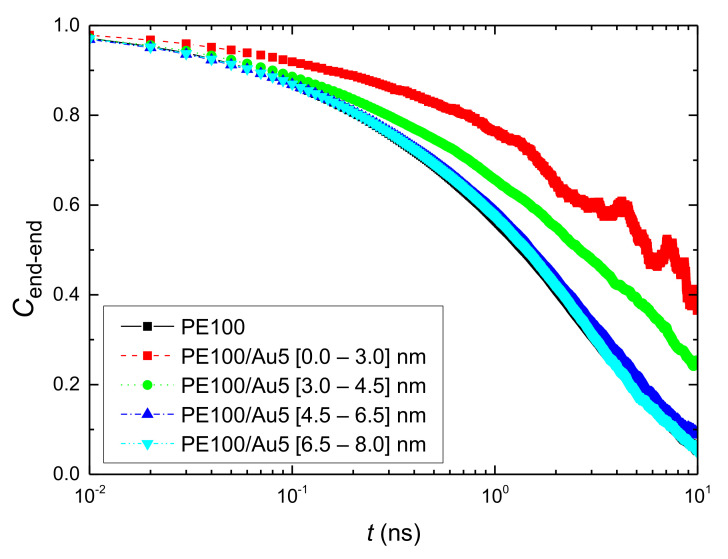
The reorientation time autocorrelation function (ACF) *C*_end-end_(*t*) as a function of time for the end-to-end vector of polyethylene for PE100/Au5 system. PE chains are analyzed across various shells from the Au NP.

**Figure A6:**
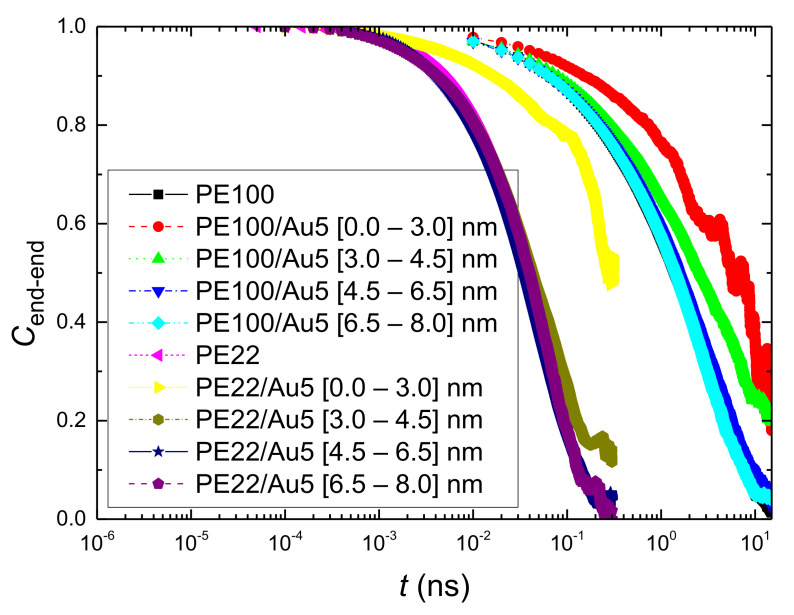
Time ACF of the end-to-end vector of PE chains, *C*_end-end_(*t*), as a function of time for PE22/Au5 and PE100/Au5 systems. *C*_end-end_(*t*) values for the PE22/Au5 and PE100/Au5 systems, for various spherical shells are presented.

First, we study the orientational dynamics at the terminal level, via the reorientation time autocorrelation function (ACF) of the end-to-end vector, defined as:

(4) $$C_{\mathrm{end}-\mathrm{end}}\left(t\right)=\frac{\langle R\left(t\right)\cdot R\left(0\right)\rangle }{\langle \|R{\|}^{2}\rangle }$$

where *R*(*t*) and *R*(0) is the end-to-end vector at time t and 0 respectively, ‖*R*‖ is its magnitude, and 〈 〉 denotes statistical average. Results for the autocorrelation function, *C*_end-end_(*t*) at different radial adsorption layers are presented in Figure 7 for the hybrid PE100/Au5 system and the comparison with PE22/Au5 system in Appendix A Figure A6. In these figures corresponding data for a bulk PE system are also shown. It should be noted that we monitored the position of each vector only for the time period it belongs to the corresponding analysis regime in order to make these calculations. It is clear that in all systems slower PE chain dynamics at the vicinity of the Au nanoparticles is shown. In particular, PE chains in the first adsorption layers show much slower terminal dynamics compared to the bulk one. Then moving away from the Au NP surface up to a specific distance, we observed a more rapid decorrelation, whereas beyond this all curves coincide. We’ve also calculated the average value of the ACF for the entire system, which is almost identical with the bulk’s one.

The effect of the PE/gold nanoparticle interface on the PE terminal dynamics of each system can be further quantified by computing the corresponding chain relaxation times, through proper fits of curves shown in Figure 7, with a Kohlrausch–Williams–Watts (KWW) stretch exponential function [128] of the form:

(5) $$C_{\mathrm{end}-\mathrm{end}}\left(t\right)=\exp\left[-{\left(\frac{t}{\tau_{\mathrm{KWW}}}\right)}^{\beta }\right]$$

where, *τ*_KWW_ is the KWW relaxation time and *β* the stretch exponent, which describes the broadness of the distribution of the relaxation times (i.e., the deviation from the ideal Debye behavior *β* = 1). Then, the relaxation time, *τ*_end-end_, is calculated as the integral of the KWW curves through:

(6) $$\tau_{\mathrm{end}-\mathrm{end}}=\frac{\tau_{\mathrm{KWW}}}{\beta }\Gamma \left(\frac{1}{\beta }\right)$$

where *Γ*( ) is the gamma function.

**Figure 8:**
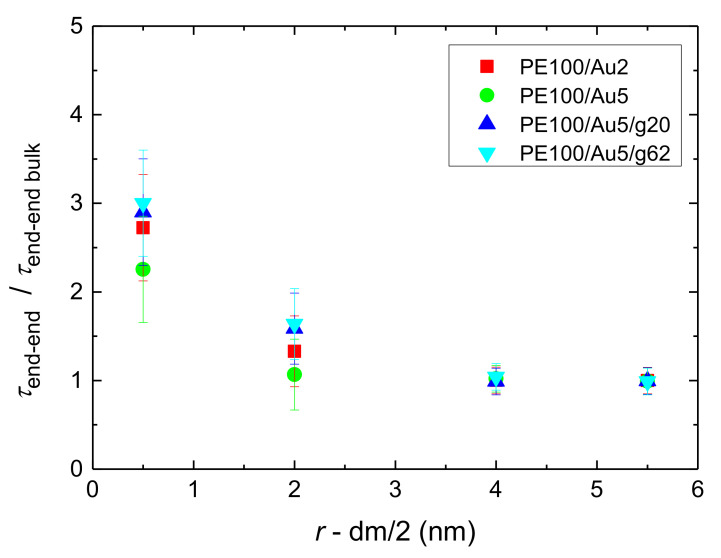
Relaxation time of the end-to-end vector of PE chains, scaled with the value of bulk chains, *τ*_end-end_*/τ*_end-end bulk_, as a function of distance from the center of the Au NP, *r*, minus the half diameter of the NP, for all systems with PE matrices consisting of 100 mers per chain.

**Figure 9:**
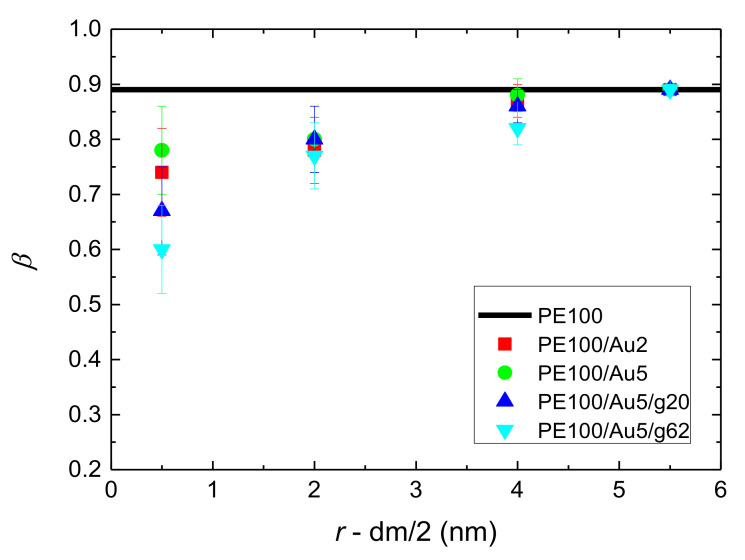
The stretch exponent *β*, as extracted from the fit with KWW functions, of the end-to-end vector ACF, *C*_end-end_(*t*), as a function of distance from the center of the Au NP, *r*, minus the half diameter of the NP for all systems with PE matrices consisting of 100 mers per chain. Black lines represent *β* values of bulk PE chains.

**Figure A7:**
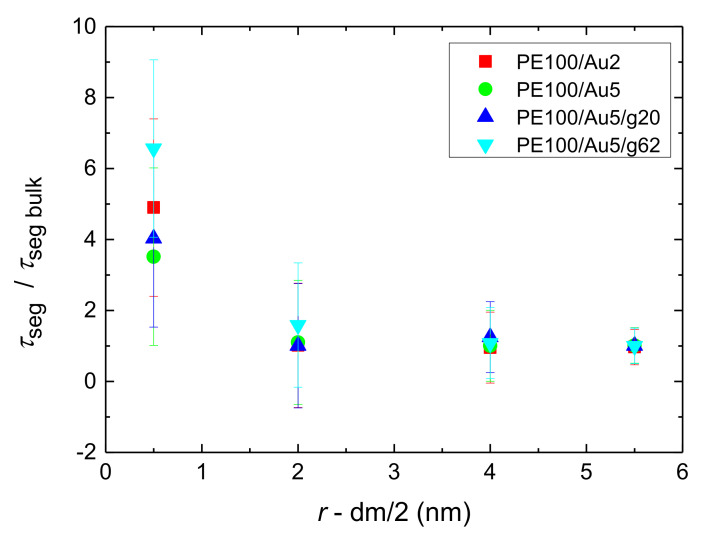
Segmental relaxation times of PE chains, scaled with the value of bulk chains, *τ*_seg_*/τ*_seg bulk_, as a function of distance from the center of the Au NP, *r*, minus the half diameter of the NP, for all systems with PE matrices consisting of 100 mers per chain.

**Figure A8:**
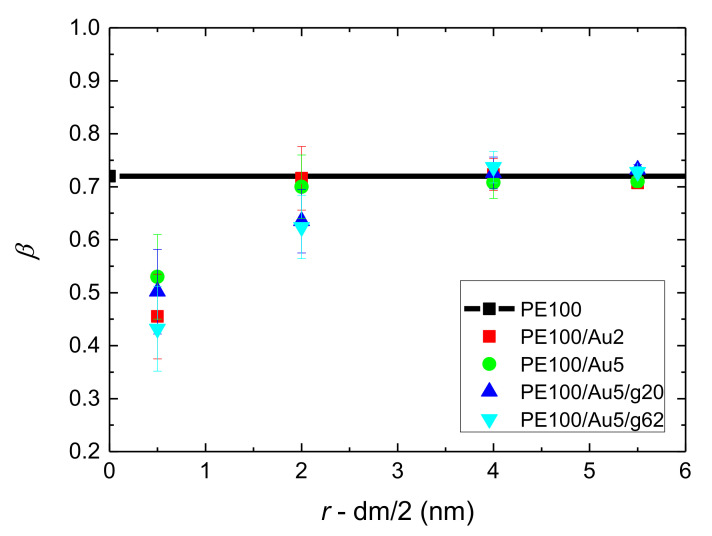
The stretch exponent *β*, as extracted from the fit of *C*_1–3_(*t*) ACF with a KWW, as a function of distance from the center of the Au NP, *r*, minus the half diameter of the NP, for all systems with PE matrices consisting of 100 mers per chain. Black lines represent *β* values of bulk PE.

**Figure A9:**
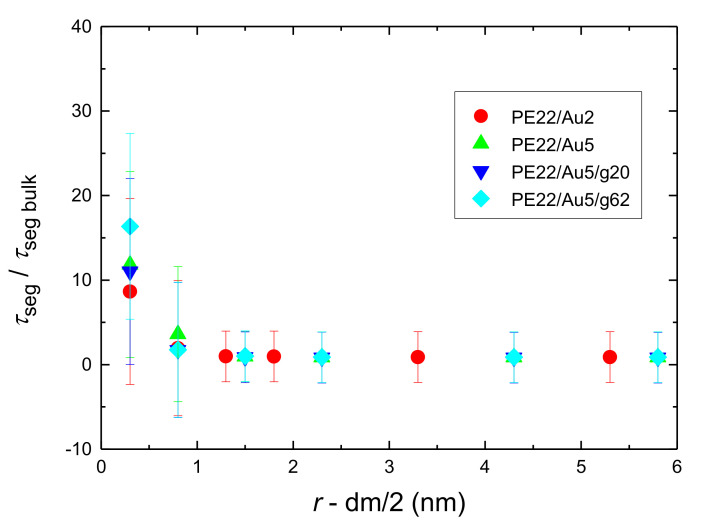
Segmental relaxation times of PE chains, scaled with the value of bulk chains, *τ*_seg_*/τ*_seg bulk_, as a function of distance from the center of the Au NP, *r*, minus the half diameter of the NP, for all systems with PE matrices consisting of 22 mers per chain.

**Figure A10:**
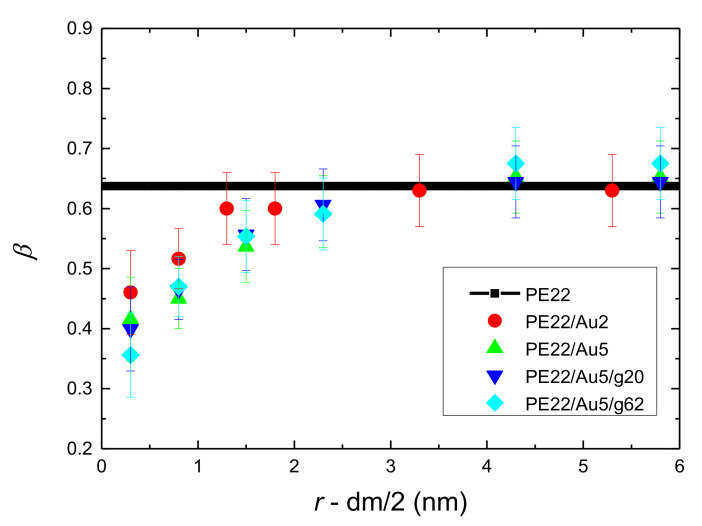
The stretch exponent *β*, as extracted from the fit of *C*_1–3_(*t*) ACF with a KWW, as a function of distance from the center of the Au NP, *r*, minus the half diameter of the NP, for all systems with PE matrices consisting of 22 mers per chain. Black lines represent *β* values of bulk PE.

**Figure A11:**
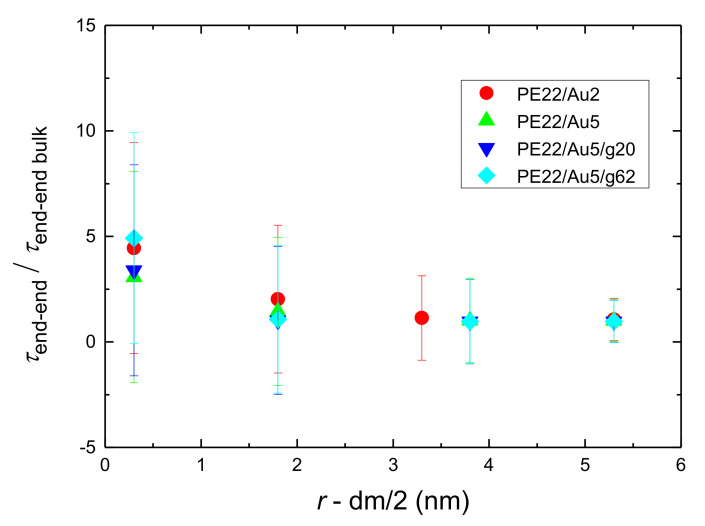
Terminal relaxation times of PE chains derived for the end-to-end vector ACF, *C*_end-end_(*t*), scaled with the value of bulk chains, *τ*_end-end_/*τ*_end-end bulk_, as a function of distance from the center of the Au NP, *r*, minus the half diameter of the NP for all systems with PE matrices consisting of 22 mers per chain.

**Figure A12:**
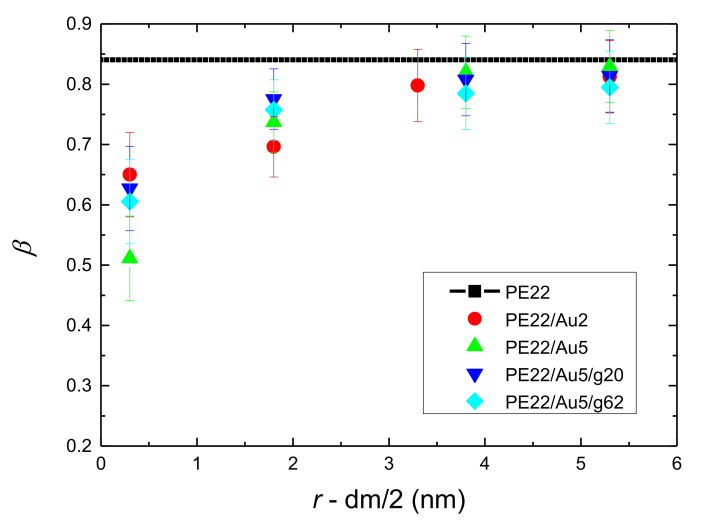
The stretch exponent *β*, as extracted from the fit with KWW functions, of ***v***^end−end^ characteristic vector based on *C*_end-end_(*t*) time autocorrelation as a function of *r* (distance from the center of the Au NP) minus the half diameter of the NP, for all systems with PE matrices consisting of 22 mers per chain. Black lines represent *β* values of bulk PE.

The results of the above analysis for both the relaxation time *τ*_end-end_ and the *β* exponent for PE chains of all the simulated systems are presented in Figure 8 and Figure 9. Bulk values are also shown in these figures. It is clear that the PE chains which are very close to the Au NP, have much slower orientational dynamics (longer terminal relaxation time *τ*_end-end_) and *τ*_end-end_ is about 2–10 times longer than the bulk one. As expected polymer chains become more mobile as their distance from the gold nanoparticle increases, reaching a plateau, bulk-like regime, at distances of about 2.5–3.0 nm away from the Au NP. From the relaxation times reported in Figure 8 it is clear that the adsorbed polymer chains are (several times) slower than the ones in the bulk-like regime, however they are still mobile, as it is also shown below by probing the translational dynamics of polymer chains. In addition, *β*-exponent values of PE chains are smaller than the bulk value (~0.89), the black line shown in Figure 9, at the majority of all distances. The latter indicates a broader distribution of the polymer terminal dynamics, compared to the bulk one. Furthermore as was expected, the 100 mers PE systems have much slower relaxation times in comparison to those of the 22 mers PE systems (Appendix A Figure A7, Figure A8, Figure A9, Figure A10, Figure A11 and Figure A12).

#### 3.2.2. Translational Dynamics

**Figure 10:**
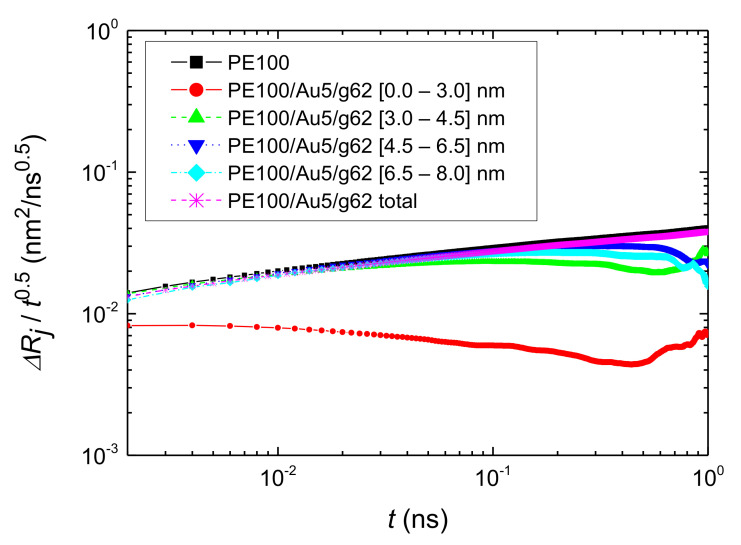
Segmental MSD of PE chains along *r* (distance from the center of the gold NP), *ΔRj*, scaled with *t*^0.5^ Data for the PE100/Au5/g62 system, for various spherical shells, and the total MSD of the PE100 and PE100/Au5/g62 systems are shown.

**Figure A13:**
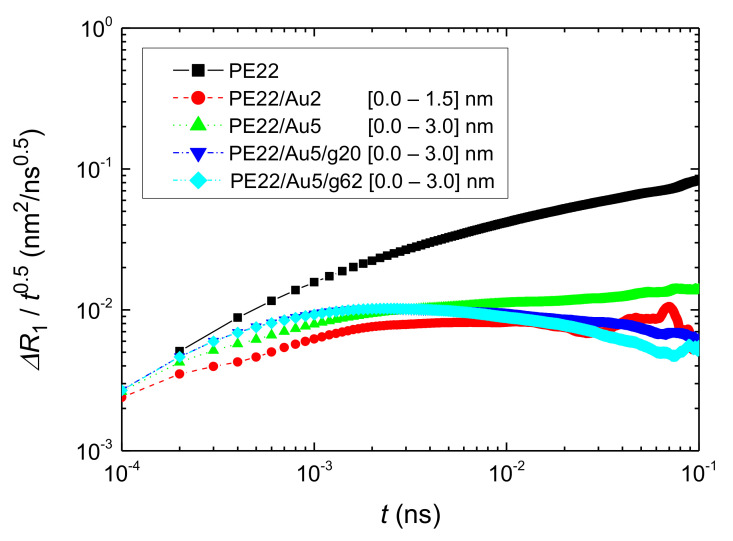
Segmental MSD of PE chains for the first adsorption spherical shell, *ΔR*_1_, scaled with *t*^0.5^. Data for the PE22/Au2, PE22/Au5, PE22/Au5/g20 and PE22/Au5/g62 systems are shown, together with data for the bulk PE22 system.

Next, the translational segmental dynamics of PE chains was examined. We have calculated the average segmental mean square displacement (MSD) in order to distinguish translational dynamics for different layers. The average segmental MSD is defined as:

(7) $$\Delta R_{j}\left(\tau \right)={\langle \left[r\left(t+\tau \right)-r\left(t\right)\right]}^{2}\rangle$$

where *j* is a specific radial region, *r*(*t*) and *r*(*t + τ*) are the coordinate vectors of a segment (CH_2_ or CH_3_ group here) within region *j*, at time *t* and *t + τ*, respectively, and brackets 〈 〉 denote statistical average for all segments within the region *j*. Note, that in the analysis used here a segment contributes to the above MSD for a given time interval *τ* and for a radial region *j*, if and only if it was constantly present in that region in the entire course of time *τ*. Data on Δ*R_j_(τ)* for all (radial) adsorption layers, scaled with *t*^0.5^, for the PE100/Au5/g62 system is shown in Figure 10. We observed slower terminal dynamics of the polymer atoms closer to the Au NP atoms (mainly in the first adsorption layer) in comparison to the one of the atoms in the other layers. In contrast, chains which belong to the other regimes, (above the second layer) show quite similar dynamics, almost equal to the bulk one, the black line and the total average value of the entire system, the magenta line shown in Figure 10. All the simulated hybrid systems have a similar behavior. However, the PEs in PE22/Au2, PE22/Au5, PE22/Au5/g20 and PE22/Au5/g62 (see Appendix A Figure A13) are faster than the equivalent systems with PE matrices consisting of 100 mers per chain.

According to the Rouse model predictions [69] Δ*R_j_*(*τ*) ∝ *τ*^1/2^. Our calculations using the data for bulk PE (PE100 system) showed that the Rouse regime was well-attained for the linear bulk chains, as it has been shown also in previous works [129,130,131]. Concerning the different adsorption spherical shells we extracted exponents less than 1/2. Those exponents indicate the variation from the Rouse behavior which is more pronounced close to the Au NP. This attributed to the fact that there is attraction of the PE monomers from the Au NP and from the grafted polymers. Furthermore, according to our analysis method, we calculated the MSD for the hybrid systems as long as the segments were within the spherical shells. Therefore the time frame window is not enough to reach the Rouse regime for the PE monomers that are close to the surface of the Au NP.

**Figure 11:**
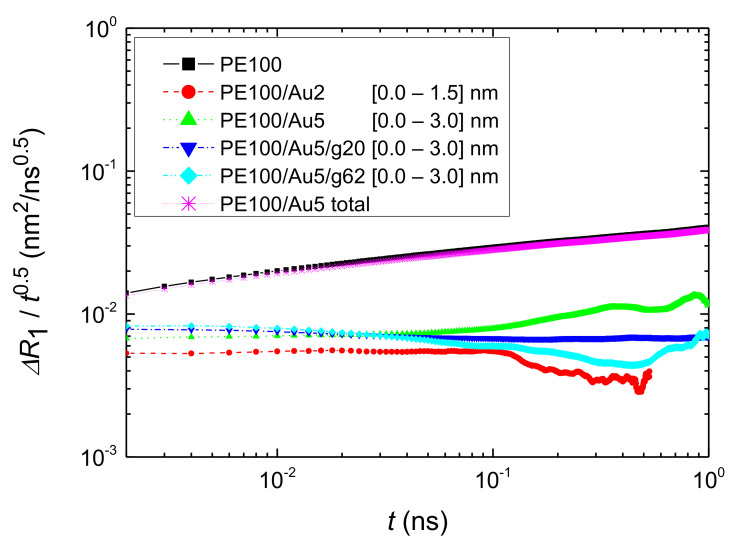
Segmental MSD of PE chains for the first adsorption spherical shell, *ΔR*_1_, scaled with *t*^0.5^. Data for the PE100/Au2, PE100/Au5, PE100/Au5/g20 and PE100/Au5/g62 systems are shown, together with data about the MSD of the entire PE100 and PE100/Au5 systems.

The MSD at the 1st adsorption spherical shell, Δ*R*_1_*(τ),* scaled with *t*^0.5^, is presented in Figure 11 for all simulated systems with PE matrices consisting of 100 mers per chain. We observe that the MSD, Δ*R_j_(τ)* in all systems for the 1st adsorption shell is smaller than the corresponding bulk one. Nevertheless, in qualitative agreement with the orientational segmental dynamics discussed above, chains in the first adsorption layer are still mobile.

## 4. Discussion and Conclusions

The term of interphase, a three-dimensional zone, is used to indicate a regime between two phases where properties are gradually changing from one phase to another, in contrast to the more traditional two-dimensional like “interface” one. For polymer nanocomposites the properties of polymer chains at the interphase are of paramount importance for the performance of these composite systems. Here, we studied, through detailed atomistic MD simulations, polymer nanocomposites with bare and core/shell metal (gold) NPs. Polyethylene chains of different molecular weights, consisting of 20 monomers per chain and 100 monomers per chain are used as a model polymer matrix. Au NPs were constructed in their equilibrium Wulff shape (minimum surface free energy) via DFT calculations. Two different Au NP sizes of 2.5 nm and 5.0 nm diameter were modeled. For the functionalized NPs grafted anchors of 20 and 62 monomers per anchor chain were considered. Various structural and dynamical properties of the PE chains were computed to examine the structure and the width of the PE/Au NP interphase.

The behavior of polyethylene is affected by the spatial heterogeneities induced by the presence of PE/Au NP interfaces. The average properties of the hybrid systems are, as expected, close to those of the bulk PE, due to the rather low concentration of the NPs. However, the dynamical properties of the polymers show big changes near the Au NPs as the relaxation times are 3 times higher than the bulk values. A detailed analysis was proposed based on averaging over atoms (or chains) within radial spherical shells equidistant from the center of the gold NP which allows us to examine the way that spatial heterogeneities are related to structural and dynamical features of the hybrid system as a function of distance from the polymer/gold nanoparticle interface.

From the analysis of the simulation it is clear that the width of the PE/Au NP interphase depends on the actual property under study. First, at the vicinity of the bare Au NPs a dense layer of polymer atoms appears. Second, for the case of the core/shell NPs the anchors change polymer’s behavior/properties and especially the density profile. Furthermore, dynamics become slower close to the Au NP and terminal relaxation time decreases with the distance from the Au NP. Both NPs exhibit qualitatively the same influence on the segmental dynamics of PE chains. Nevertheless, there is a difference on the region defined from the center of the NP that is affected: It is about 2.5 nm for the small NP and about 4.0 nm for the larger one and as expected, the bigger the NP the larger the percentage of PE chains that are affected. Finally, the examined polymer properties attain their bulk-like values far from the Au NP’s surface, whereas as expected by increasing the molecular weight, the density (slightly) increases and the dynamics become slower.

In Table 2 we summarize the effect of interface on various properties. Results can be summarized as follows:

- Local structural and conformational features were analyzed at the level of both individual segments (atoms or bonds) and entire chains. Due to the intermolecular PE/Au NP (adhesive) interaction the local monomer PE mass density exhibits a maximum near the gold surface. At short distances chain segments tend to orientate almost parallel to the Au NP surface. This randomizes gradually as the chain segments move away from the interface. Furthermore, in the dihedral angle distribution at the PE/gold NP interface we observed an increase of “trans” population compared to the bulk one. This reflects the more ordered polymer chain structures.
- Orientational relaxation of PE chains in the hybrid systems at the segmental and terminal level was quantified through the time autocorrelation function of a segmental vector and the end-to-end vector of PE chain respectively. In all cases the PE chains which were closer to the Au NP had much slower orientantional dynamics (segmental relaxation time, *τ*_seg_, is about 10 times longer) in comparison to the bulk one. Moving away from the interface up to a specific distance, we noticed faster *C*_1–3_(*t*) decorrelation, while beyond this, all curves coincide. Moreover, we observed broader distribution of the polymer orientational dynamics in comparison to the bulk one (smaller *β*-exponent values).
- Translational segmental and center of masses dynamics of PE chains were examined by calculating the average mean-square displacement. Due to the polymer/gold nanoparticle interfaces, for all model hybrid systems, PE chains closer to the Au NP are slower, compared to the bulk one.

The above findings, beside their obvious relevance to scientific questions regarding the influence of metal (Au) NPs on the properties of the polymer matrix might have also implications to technological applications, as the properties of the polymer/metal interface affect the functional properties of the hybrid material. As an example, polymer mass density profiles are directly related to the actual polymer/NP interaction.

From the technological point of view, an interesting aspect concerns the comparison of the different model systems studied in this work, as candidates for given technological applications. This is a non-trivial issue, since which model system is the “best one” depends strongly on the specific application. In general, for cases where strong interaction between polymer and metal NPs is necessary PE/Au nanocomposites (NCs) with bare NPs are advantageous. On the contrary, for cases were better dispersion of the Au NPs and more theta-solvent like polymer-Au interactions are desired, PE/Au NCs with core-shell Au NPS are a better choice.

Polymer nanocomposites have been studied via molecular simulations. However, limited are the works concerning PNCs with metal NPs. The results presented here are a first step towards a fundamental understanding of specific polymer/metal (here PE/Au) nanocomposites via detailed atomistic simulations. To achieve this, additional works providing systematic investigations of the role of chemistry and of specific interactions on the properties of polymer/metal nanocomposites are required. Examples include PNCs with metal NPs of different size and shape, of different polymer matrix (e.g., conductive polymers), or systems with core-shell NPs of different grafting density.

Another interesting issue concerns the dispersion of the metal (Au) NPs in the polymer matrix, which depends strongly on the actual polymer/NP and NP/NP interactions. In this work a single NP has been used, so it is not possible to directly study the dispersion of Au NPs. However the polymer mass density profiles at the interface is a direct evidence of the polymer/NP interaction. From these data we can speculate that the interaction of PE chains with the core-shell Au NPs is more like a theta solvent, since the hair have the same chemistry as the matrix. Therefore, we would expect that these NPs could disperse better in the polymer matrix than the bare ones [132]. Moreover, we can assume that the longer the grafted chains and the higher the grafting density the better the dispersion of the core-shell Au NPs in the PE matrix. This could be further studied by calculating the potential of mean force between two NPs. All of them are potential directions for future work.

## Figures and Tables

**Table 1:** Details of the simulated model systems.

Name	Au NP Diameter	Au Atoms	Free PE Chains	Au/PE *w*/*w*%	Au/PE *v*/*v*%	Grafted PE Chains	Grafted PE Mers/Chain
PE100/Au2	25.1 Å	459	1200	4.9	0.2	-	-
PE100/Au5	50.2 Å	3101	1200	37.6	1.7	-	-
PE100/Au5/g20	50.4 Å	2461	1200	29.7	1.7	53	20
PE100/Au5/g62	50.4 Å	2461	1200	29.7	1.7	53	62
PE100	-	-	240	-	-	-	-
PE22/Au2	25.1 Å	459	5040	5.8	0.4	-	-
PE22/Au5	50.2 Å	3101	5040	38.8	1.6	-	-
PE22/Au5/g20	50.4 Å	2461	5040	30.8	1.6	53	20
PE22/Au5/g62	50.4 Å	2461	5040	30.8	1.6	53	62
PE22	-	-	420	-	-	-	-

**Table 2:** The width of the interface in PE/au NP nanocomposites defined via different properties for the systems with 100 mers PE chains.

Property	Bare Au NPs	Grafted Au NPs
Density	0.5–1.0 nm	1.7–3.0 nm
Structural	0.5–1.0 nm	0.5–1.3 nm
Local (segmental) dynamics	1.0–2.0 nm	0.5–1.5 nm
Global dynamics	3.0–4.0 nm	1.0–2.0 nm

## Data Availability

The data presented in this study are available on request from the corresponding author.

## Supplementary Materials

The following is available online at https://www.mdpi.com/2073-4360/13/4/541/s1, Video S1: PE-grafted AuNP. Video from MD simulation of hybrid polyethylene/grafted gold nanoparticle at 450 K. Au nanoparticle (2461 atoms, diameter of 5.04 nm) and polyethylene (1200 chains, 100-mers per chain) are shown. In yellow is the Au and in grey are the edges of Au nanoparticle. In blue are the free CH_2_ and the free CH_3_ monomers. In red are the grafted CH_2_ and CH_3_ monomers.

## Appendix A

**Table A1:** Non-bonded interactions of the atomistic force field for the model PE/Au nanocomposites.

Non-Bonded Interactions				
$V_{LJ}\left(r_{ij}\right)=4\varepsilon_{ij}\;\left[{\left(\frac{\sigma_{ij}}{r_{ij}}\right)}^{12}-{\left(\frac{\sigma_{ij}}{r_{ij}}\right)}^{6}\right]\;,\;r\leq \;R_{c}\;\;$ **Lennard-Jones**				
**Atom Types**		**mass (g/mol)**	**σ (nm)**	**ε (kJoule/mol)**
CH_2_		14.027	0.395	0.3824
CH_3_		15.035	0.395	0.3824
S–CH_2_		32.066–14.027	0.372	0.7219
S–CH_3_		32.066–15.035	0.372	0.8761
$V_{Morse}\left(r_{ij}\right)=D_{0}\;\left[e^{-2a\left(r-r_{0}\right)}-2e^{-a\left(r-r_{0}\right)}\right]\;,\;r\leq \;R_{c}\;\;$ **Morse**				
**Atom Types**	**mass (g/mol)**	**D_0_ (kJoule/mol)**	**α (nm^−1^)**	**r_0_ (nm)**
Au–CH_2_	196.967–14.027	1.6885	11.69	0.4085
Au–CH_3_	196.967–15.035	1.6885	11.69	0.4085

**Table A2:** Bonded interactions of the atomistic force field for the model PE/Au nanocomposites.

Bonded Interactions					
$V_{b}\left(r_{ij}\right)=\frac{1}{2}\;k_{ij}^{b}\;{\left(r_{ij}-b_{ij}\right)}^{2}$					
**Bond**	**b (nm)**			**k^b^ (kJ/mol·nm^2^)**	
CH_2_–CH_2_	0.154			100,000.00	
CH_2_–CH_3_	0.154			100,000.00	
CH_3_–CH_2_	0.154			100,000.00	
S–CH_2_	0.181			fixed	
$V_{\alpha }\left(\theta_{ijk}\right)=\frac{1}{2}k_{ijk}^{\theta }{\left(\theta_{ijk}-\theta_{ijk}^{0}\right)}^{2}$					
**Angle**	**θ° (deg)**			**k^θ^ (kJ/mol * rad^2^)**	
CH_2_–CH_2_–CH_2_	114			519.611	
CH_3_–CH_2_–CH_2_	114			519.611	
CH_2_–CH_2_–CH_3_	114			519.611	
S–CH_2_–CH_2_	114			519.611	
$V_{opls}\left(\varphi_{ijkl}\right)=\frac{1}{2}{Κ}_{1}\left[1+\cos\left(\varphi \right)\right]+\frac{1}{2}{Κ}_{2}\left[1-\cos\left(2\varphi \right)\right]+\frac{1}{2}{Κ}_{3}\left[1+\cos\left(3\varphi \right)\right]+\frac{1}{2}{Κ}_{4}\left[1-\cos\left(4\varphi \right)\right]\;$					
**Dihedral**	${Κ}_{1}$ **(KJ/mol)**	${Κ}_{2}$ **(KJ/mol)**	${Κ}_{3}$ **(KJ/mol)**		${Κ}_{4}$ **(KJ/mol)**
CH_3_–CH_2_–CH_2_–CH_2_	4.276	−1.12968	13.1545		0.00
CH_2_–CH_2_–CH_2_–CH_2_	4.276	−1.12968	13.1545		0.00
CH_2_–CH_2_–CH_2_–CH_3_	4.276	−1.12968	13.1545		0.00
S–CH_2_–CH_2_–CH_2_	4.276	−1.12968	13.1545		0.00

Below in Figure A6, Figure A7, Figure A8 and Figure A9 data about the segmental dynamics of the PE chains are shown. We follow a methodology similar to the one for the end-to-end vector. First, we compute the angle *θ* of a segmental 1–3 vector, ***v***^1−3^, at time *t* relative to its position at *t* = 0. Second, we calculate the second order Legendre polynomial (correlation function) for this vector, defined as:

(A1) $$C_{1-3}\left(t\right)=\frac{3}{2}\left(\langle \cos\theta \left(t\right)\cos\theta \left(0\right)\rangle -\frac{1}{2}\right)$$

Then, we fit *C*_1–3_(*t*) using a KWW function and derive the characteristic segmental relaxation time, *τ*_seg_ and the corresponding *β*-exponent, by computing the integral below the KWW curve, similar to the analysis followed for the end-to-end vector ACFs.
